# Epigenetic Regulation of Adipogenesis in Development of Metabolic Syndrome

**DOI:** 10.3389/fcell.2020.619888

**Published:** 2021-01-12

**Authors:** Richa Pant, Priyanka Firmal, Vibhuti Kumar Shah, Aftab Alam, Samit Chattopadhyay

**Affiliations:** ^1^National Centre for Cell Science, SP Pune University Campus, Pune, India; ^2^Roswell Park Comprehensive Cancer Center, Buffalo, NY, United States; ^3^Department of Biological Sciences, BITS Pilani, Goa, India

**Keywords:** obesity, adipogenesis, insulin resistance, metabolic syndrome, transgenerational inheritance

## Abstract

Obesity is one of the biggest public health concerns identified by an increase in adipose tissue mass as a result of adipocyte hypertrophy and hyperplasia. Pertaining to the importance of adipose tissue in various biological processes, any alteration in its function results in impaired metabolic health. In this review, we discuss how adipose tissue maintains the metabolic health through secretion of various adipokines and inflammatory mediators and how its dysfunction leads to the development of severe metabolic disorders and influences cancer progression. Impairment in the adipocyte function occurs due to individuals’ genetics and/or environmental factor(s) that largely affect the epigenetic profile leading to altered gene expression and onset of obesity in adults. Moreover, several crucial aspects of adipose biology, including the regulation of different transcription factors, are controlled by epigenetic events. Therefore, understanding the intricacies of adipogenesis is crucial for recognizing its relevance in underlying disease conditions and identifying the therapeutic interventions for obesity and metabolic syndrome.

## Introduction

Obesity is defined as excessive or abnormal fat accumulation in the body which may impair the health of an individual. Body mass index (BMI), which is considered the most simple and useful index of weight-for-height in the entire world, provides only a rough estimate to categorize people with obesity in adult population^1^. Therefore, the concept of metabolically healthy obesity and metabolically unhealthy obesity is gaining attention as in addition to gaining abdominal weight, hormonal and metabolic profile of an individual also counts (Naukkarinen et al., 2014). The increasing incidences of obesity ignited a huge interest in understanding the process promoting efficient energy storage and curtailing the adverse metabolic consequences of obesity such as diabetes, hypertension, dyslipidemia, atherosclerosis and fatty liver diseases. The ability of adipocytes to effectively store lipids prevents the toxic lipid accumulation in other organs. In fact, adipose tissue can expand in response to excess lipid accumulation to maintain the energy homeostasis (Wang et al., 2013) but the capacity of adipose tissue to store fat or to expand in response to fat storage is limited. Once exceeded, lipids might spill into other organs that are not suitable for fat storage resulting in insulin resistance (IR) and other metabolic complications (Tan and Vidal-Puig, 2008). Alteration in fat mass also results in alteration in adipokine profile of an individual. Obesity is linked with an increase in leptin concentration and a decrease in adiponectin levels (Matsubara et al., 2002). In addition to these two prototype adipokines, many other factors are known to get altered in obesity. Obese state is also identified by an increased macrophage infiltration in the adipose tissue. These macrophages and other immune cells infiltrated in the adipose tissue are a source of TNF-α, IL-6, and other cytokines that links obesity with inflammation and IR (Weisberg et al., 2003). Altered immune response and adipokine secretion are also known to increase the risks of certain cancers such as breast, ovarian, kidney, endometrial, colorectal, etc. (see text footnote 1). Past few decades have shown some great advancement in understanding transcriptional and epigenetic regulation of adipogenesis. Peroxisome-proliferator activator receptor γ (PPARγ) and CCAAT/enhancer binding protein α (C/EBPα) are the two key transcription factors which regulates the adipocyte formation. They work in co-ordination with transcriptional co-activators and epigenetic regulators modulating the gene expression profiles during adipocyte differentiation (Madsen et al., 2014). Advancement in molecular biology techniques unfolded the key mechanisms of epigenomic regulation during adipogenesis and revealed the significance of histone modification, DNA methylation, and chromatin remodeling in adipocytes differentiation (Lee et al., 2019). These epigenetic changes are influenced by certain environmental factors such as energy-rich foods, changes in sleep cycle, sedentary lifestyle, medicated drugs and environmental chemicals that have potential to reprogram the epigenetic patterns and induce adiposity (Gangwisch et al., 2005; McAllister et al., 2009). The environment-epigenetic interaction also results in transgenerational and lifestyle-induced obesity (Youngson and Morris, 2013). Moreover, exposure to high fat diet and environmental toxins *in utero* may affect the metabolic outcomes in future generations through epigenetic transgenerational inheritance of obesity (Vandegehuchte et al., 2010; Dunn and Bale, 2011).

This review focuses on the interplay between adipose tissue function, adipokines, systemic inflammatory profile and metabolic health. We have also discussed the transcriptional and epigenetic regulators involved in adipogenesis and their interaction with environment responsible for transgenerational inheritance of the disease. Understanding the molecular mechanism of adipogenesis and the complexities associated with it will help in finding the plausible therapeutic approaches for treatment of obesity.

## Adipose Tissue: White, Brown and More

Adipose tissue is a loose connective tissue which is critical in regulating energy metabolism, i.e., energy storage and expenditure. Adipocytes or the fat cells contribute around 35–70% of adipose tissue mass in an adult human. Besides adipocytes, some other cell types like macrophages, blood cells, fibroblasts, endothelial cells, etc., are also present in the adipose tissue (Frühbeck, 2008). Morphologically, adipose tissue can be classified into three types: white, brown and beige. White adipose tissue (WAT) is mostly composed of unilocular adipocytes and its key function is to store surplus energy as triglycerides during excess nutrient condition. The stored triglycerides are utilized for energy generation under energy deficit conditions such as fasting, exercise or prolonged food deprivation (Li et al., 1993; Blüher, 2013). On the other hand, brown adipose tissue (BAT) consists of mitochondria-rich multilocular adipocytes. The main function of brown adipose tissue is to dissipate energy in the form of heat through mitochondrial uncoupling upon β-adrenergic stimulation (Foster and Frydman, 1979). BAT was formerly believed to have functional role in rodents, hibernating mammals, and partly in human infants but recently, adult humans have shown functional BAT upon mild cold exposure and activation of sympathetic nervous system (Cypess et al., 2013, 2015). β3-adrenergic receptor (β3-AR) agonist can stimulate human BAT thermogenesis and help in treatment of obesity and metabolic diseases (Cypess et al., 2015). A clinical trial of β3-AR agonist, mirabegron, stimulated BAT metabolic activity and increased WAT lipolysis in human subjects (Baskin et al., 2018). In addition to white and brown fat, there also exists a third type known as beige/brite fat. As the name implies, brite fat is the accumulation of brown adipocytes within the white fat depots. Beige cells have the unique ability to shift between energy storage and energy expenditure phenotype (Wu et al., 2012). A study by Zhang et al. demonstrated that embryo-derived white adipose stem cells (eWAsc) have excellent beige adipogenic potential. The study showed potential in widening the research on human adipocytes (Zhang et al., 2019). There also exists a functional relationship between angiogenesis and brite/beige adipocyte development. The pro-angiogenic conditions helps in proliferation of beige/brite adipocytes and transplantation of human brite adipocytes improves the systemic glucose homeostasis in diet induced obesity (DIO) mice model. Since brite adipocytes were found to enhance glucose homeostasis, they could be implied to have potential therapeutic benefits (Min et al., 2016).

**FIGURE 1:**
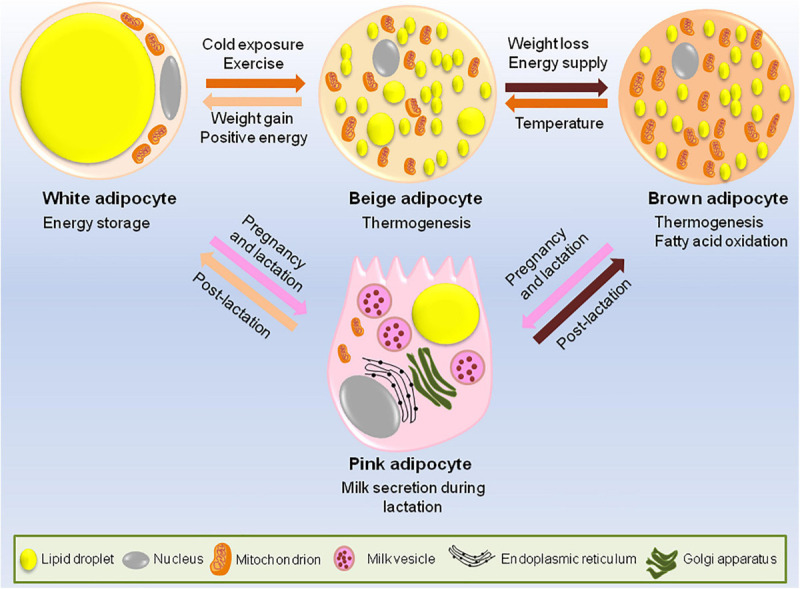
Adipocytes have remarkable plastic properties. In usual scenario adipose tissue consists of white, brown and occasional beige adipocytes. The main function of white adipocytes is to store lipids to meet the metabolic requirements of the body while brown adipocytes are required for thermogenesis. Beige adipocytes have the ability to switch between energy storage and expenditure. However, during certain conditions like cold exposure or strenuous exercise, white adipocytes trans-differentiates to beige or brown adipocytes while during the state of positive energy when there is lack of lipid storage, brown/beige adipocytes can be converted back to white adipocytes to increase the energy stores. During pregnancy and lactation, subcutaneous white adipocytes of the breast tissue convert to pink adipocytes which are basically the milk secreting glands formed by lipid-rich elements and brown adipocytes trans-differentiate to myoepithelial cells of mammary glands. All these conversions are reversible, i.e., post-lactation, pink adipocytes convert back to white and brown adipocytes.

Adipocytes have an astonishing plastic property, i.e., white adipocytes can trans-differentiate to brown adipocytes. In fact, during pregnancy and lactation, white adipocytes specific to mammary gland convert reversibly to milk producing epithelial cells (also called pink adipocytes because they appear pink at macroscopic level) and brown adipocytes trans-differentiate to myoepithelial cells (cells of alveolar glands) (Figure 1). Once the lactation period is over, pink adipocytes convert back to white and brown adipocytes (Morroni et al., 2004; Giordano et al., 2014).

The fat cells (adipocytes) develop from adipocyte precursor cells (pre-adipocytes) in a process called adipogenesis which occurs throughout the lifespan of an organism (Billon et al., 2007). The differentiation of pre-adipocytes to lipid-laden adipocytes is widely studied *in vitro.* Amongst all the studied cell lines, the most widely used ones which provide the important insights in regulating late steps of adipocyte development are 3T3-L1 and 3T3-F422A (Green and Meuth, 1974; Green and Kehinde, 1975). Mouse embryonic stem cells (mESCs) also provide an alternate system for understanding early stages of adipogenesis (Billon et al., 2007). By using these biological tools, researchers have been able to recognize the key transcription factors involved in adipogenesis and many are still in the process of being identified.

## Adipose Tissue Dysfunction in Obesity and Metabolic Diseases

Obesity is defined as excessive fat accumulation that may impair the health and wellbeing of an individual. Sedentary lifestyle, urbanization, easy affordability and accessibility to high calorie food may account for excess energy intake and weight gain within the population (Afshin et al., 2017). Apart from some parts of sub-Saharan Africa and Asia, the number of people with obesity surpasses the number of people who are underweight. Globally, this accounts for more deaths from obesity than malnutrition^2^. The development of obesity not only depends upon the balance between energy intake and expenditure but also on the balance between WAT and BAT. Unhealthy expansion of WAT is one of the major culprits contributing to obesity-associated metabolic complications.

White adipose tissue accounts for 5–50% of human body weight and has a central role in energy homeostasis (Kajimura, 2017). Anatomically, WAT can be categorized as visceral adipose tissue or VAT (intra-abdominal, surrounding the internal organs) and sub-cutaneous adipose tissue or SAT (under the skin). Amongst the two types, visceral fat is said to be strongly associated with increased metabolic risk than subcutaneous fat (Hayashi et al., 2008). Additionally, the associated risk factor is more pronounced in women than men (Fox et al., 2007). It has been observed that in 3D adipocyte-ECM culture, SAT ECM rescued the defects in glucose uptake and adipogenesis specific gene regulation in VAT adipocytes while VAT ECM impaired the adipocyte function in SAT adipocytes. This suggests the importance of extracellular matrix-adipocyte crosstalk in regulation of depot-specific adipocyte function in murine obesity and metabolic diseases (Strieder-Barboza et al., 2020).

In majority of lean and healthy individuals, WAT is mostly restricted to subcutaneous depots but in individuals with obese/overweight phenotype, WAT mass can expand ectopically in areas other than their specific depots as a result of lipodystrophy (Chait and den Hartigh, 2020). Lipodystrophy is a heterogenous group of disorder characterized by abnormal adipose tissue distribution. It can be congenital or acquired and is linked with the development of IR and related co-morbidities like type 2 diabetes (T2D), hyperglycemia, hyperlipidemia, non-alcoholic fatty liver disease (NAFLD), auto-immune hepatitis or viral hepatitis in case of human immunodeficiency virus (HIV)-associated lipodystrophy (Polyzos et al., 2019). There are essentially two mechanisms to explain the development of metabolic syndrome resulting from obesity: (a) accumulation of fat in liver and muscle or other cells of the body in addition to adipose tissue resulting in IR in these organs (Petersen and Shulman, 2006) and (b) release of adipokines and cytokines from the dysfunctional adipocytes (Saltiel, 2001; Scherer, 2006). In healthy states, these adipokines and cytokines maintain the metabolic homeostasis but in obesity, the hypertrophic adipocytes and the resident immune cells hasten the pro-inflammatory profile with altered secretion of these endocrine factors thereby contributing to metabolic diseases (Scheja and Heeren, 2019). However, not all individuals with obesity develop the associated metabolic problems. The sub-group of insulin-sensitive individuals with obesity showing normal hormonal and metabolic profiles despite of their BMIs in obese category (i.e., ≥30 kg/m^2^) are classified as having “metabolically healthy obesity” (MHO) (Naukkarinen et al., 2014). These individuals are different from those having “metabolically unhealthy obesity” (MUHO) who are characterized by accumulation of intra-abdominal fat in visceral depots (central obesity), IR, pre-disposition to diabetes and other metabolic diseases (Karelis et al., 2005; Blüher, 2010). Individuals with MHO are defined as having abdominal obesity with waist circumference >88 cm in women and >102 cm in men. They might not develop any of the risk factors such as increased fasting plasma glucose, high triglycerides, low HDL cholesterol and high blood pressure, two or more of which are observed commonly in MUHO (Grundy et al., 2005; Janiszewski and Ross, 2010).

In mice and rat models, surgical removal of visceral fat pads using lipectomy improved the insulin sensitivity, longevity and decreased tumor proliferation (Gabriely et al., 2002; Lu et al., 2012). Not only in rodent models but adipose tissue removal from the mesentery of baboons (having insulin resistance and obese phenotype) also resulted in reversal of IR and significant weight loss (Andrew et al., 2018). These studies suggest the use of lipectomy as a potential clinical tool to ameliorate obesity associated co-morbidities. In summary, adipose tissue health is utmost important for maintaining the metabolic health of an individual. Any perturbance in adipose tissue function may result in long term health ailments.

## Altered Adipokine Production and the Risk of Development of Metabolic Disorders

Adipose tissue is a metabolically active endocrine organ that secretes a range of adipokines and hormones which can have different functions in human body (Derosa et al., 2020). One of the first discoveries that recognized the role of adipose tissue as an endocrine organ was the positional cloning of obese (ob) gene and detection of its 16-KDa protein product leptin (Zhang et al., 1994). Subsequent studies revealed that daily administration of recombinant OB protein to ob/ob mice lowered their food intake, body fat percentage and serum concentration of glucose and insulin. Moreover, the energy expenditure and metabolic rate of these mice were also increased with this treatment, suggesting that OB protein stabilizes the metabolic status of ob/ob mice (Campfield et al., 1995; Halaas et al., 1995; Pelleymounter et al., 1995). Since then, leptin is known to regulate whole body metabolism through inhibiting food intake, restoring euglycemia and stimulating energy expenditure. In 2014, AstraZeneca’s myalept/metreleptin (recombinant human leptin) was approved by the United States Food and Drug Administration to treat generalized lipodystrophy (^3^ identifier: NCT00677313) (Ajluni et al., 2016). Recently, in a non-randomized crossover group study including patients with lipodystrophy, metreleptin was shown to improve insulin sensitivity and decrease circulating and hepatic triglycerides irrespective of their food intake (Brown et al., 2018). Another important protein, adiponectin, was originally described in 1995 as a 30 KDa secretory protein ‘Acrp30’ that was exclusively made in adipocytes (Scherer et al., 1995). Adiponectin functions to increase the insulin sensitivity, fatty acid oxidation and energy expenditure along with reduction in glucose production by liver (Galic et al., 2010). Adiponectin is also known to inhibit breast cancer growth by induction of cytotoxic autophagy in breast cancer cells through activation of AMPK-ULK1 axis (Chung et al., 2017). Altered adipokine production is usually associated with the risk of development of metabolic disorders. High levels of resistin and low levels of adiponectin could be predictive of future diabetic condition in people with obesity (Derosa et al., 2020). Apart from these two proteins, many different adipokines have been described in recent times that control the energy metabolism (Galic et al., 2010). An observational trial confirmed that people with obesity have higher levels of leptin, adipsin, retinol binding protein-4 (RBP-4), IL-6, high sensitivity-C reactive protein (Hs-CRP) and lower levels of adiponectin and visfatin as compared to lean people (Derosa et al., 2013). Recently, S100A4 was identified as a novel adipokine associated with IR and subcutaneous WAT inflammation/adipocytes hypertrophy irrespective of BMI although its significance as a circulating marker for dysfunctional WAT and IR is yet to be established (Arner et al., 2018). A newly discovered adipokine, asprosin, promoted the hepatic glucose release and inhibition of its activity could be used as an approach to counteract hyperinsulinism associated with metabolic disorders (Romere et al., 2016). Apelin is another adipokine which improves insulin sensitivity in humans and could be considered as a target for new therapeutic strategies to combat IR in patients with T2D (Gourdy et al., 2018). TGF-β2 is an exercise/lactate induced adipokine which improves the glucose tolerance and insulin sensitivity. HFD-fed mice treated with recombinant TGF-β2 showed reduced WAT inflammation and fat mass indicating the importance of exercise training on glucose and lipid metabolism (Takahashi et al., 2019).

**FIGURE 2:**
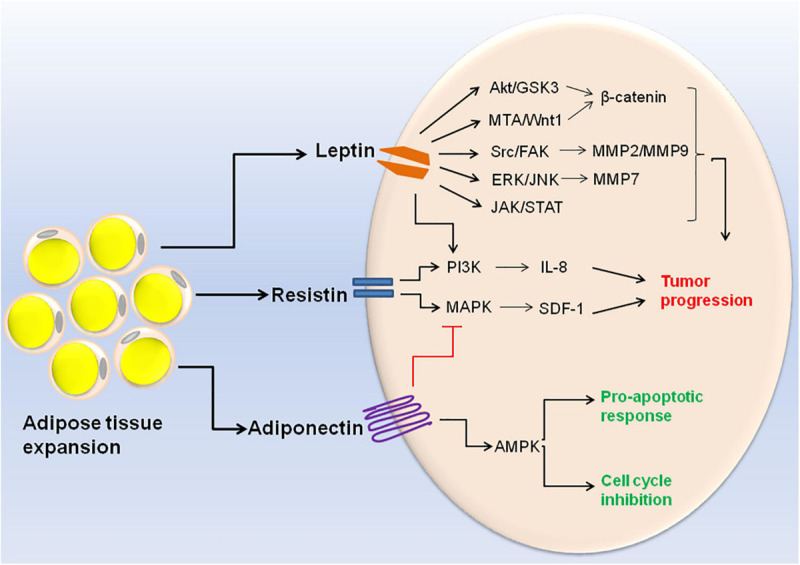
Cell signaling events triggered by altered adipokine production during obesity. Expansion of adipose tissue in obese condition leads to altered adipokine production. High concentration of leptin and resistin results in the activation of different signaling pathways within the cell (Akt/GSK3, MTA/Wnt1, Src/FAK, ERK/JNK, JAK/STAT, PI3K, and MAPK). These signaling pathways ultimately lead to cancer cell invasion and metastasis. On the other hand, high adiponectin concentration leads to MAPK inhibition and AMPK inactivation which is responsible for its pro-apototic and anti-tumoral activities.

Obesity related adipokines also play a role in etiology of different cancers. A decrease in adiponectin concentration and a corresponding increase in concentration of leptin, resistin, visfatin, IL-6, IL-8, and TNF-α are linked with progression of breast cancer (Gui et al., 2017). Moreover, decreased expression of adiponectin receptor is associated with the metastasis of human endometrioid adenocarcinoma (Yamauchi et al., 2012). Exposure of human breast cancer cell line MCF-7 to recombinant adiponectin resulted in AMPK activation and MAPK inactivation thereby inhibiting cell cycle progression. This indicates that adiponectin mediates anti-proliferative response in breast cancer cells (Dieudonne et al., 2006). Apart from adiponectin, nearly all the other adipokines exhibit pro-inflammatory and proliferative activities in cancer progression. For example resistin induces prostate cancer progression through activation of PI3K signaling pathway (Kim et al., 2011). Resistin also stimulates the expression of stromal cell-derived factor-1 (SDF-1) by activating p38 MAPK/NF-κB signaling pathway in human gastric carcinoma cells (Hsieh et al., 2014). A meta-analysis study revealed that serum leptin profile plays an important role in pathogenesis of breast cancer (Gu et al., 2019). Leptin crosstalks with various molecular mediators of the obesity such as VEGF, estrogen, IGF-1, insulin and inflammatory cytokines. Hyperactive leptin signaling potentiates these molecular mediators and leads to the activation of various oncogenic pathways resulting in enhanced proliferation and invasion of cancer cells (Saxena and Sharma, 2013). Accumulating evidences suggest that leptin induces EMT in cancer cells via different molecular pathways including JAK/STAT pathway, β-catenin activation via Akt/GSK3 and MTA/Wnt1 pathway, and activation of IL-8 via PI3K/Akt dependent pathway (Yan et al., 2012; Wang L. et al., 2015; Mullen and Gonzalez-Perez, 2016). Upregulation of pyruvate kinase muscle isozyme 2 (PKM2) along with activation of PI3K/AKT signaling can also be regarded as the potential candidate for breast cancer therapy (Wei et al., 2016). A recent research demonstrated that leptin results in the secretion of MMP2 and MMP9 in mammary epithelial cells via Src and FAK-dependent pathways (Olea-Flores et al., 2019). Leptin is also known to promote ovarian cancer invasion by inducing MMP7 expression through activation of ERK and JNK pathways (Ghasemi et al., 2018). The cell signaling events triggered by different adipokines are illustrated in Figure 2.

Leptin is often found to be associated with drug resistance. Tumor leptin expression in gastro-oesophageal adenocarcinomas is associated with resistance to cytotoxic chemotherapy (Bain et al., 2014). Additionally, leptin receptor-positive glioblastoma cells were found to be temozolomide (TMZ)-resistant (Han et al., 2013). Also, the high circulating leptin concentration could counteract cisplatin-induced cytotoxicity in breast cancer cells (Nadal-Serrano et al., 2015). Therefore, the use of non-toxic leptin antagonists that interferes with leptin signaling could serve as a novel mechanism to target leptin-induced cancers (Candelaria et al., 2017).

In addition to WAT, recent studies also reported the contribution of BAT to release secretory molecules called ‘batokines’ which make BAT functionally similar to an endocrine organ. Fibroblast growth factor 21 (FGF21), IL-6, neuregulin-4 (NRG-4), and bone morphogenetic protein-8b (BMP8b) are amongst the first few batokines to be identified. The BAT-released endocrine factors can target peripheral tissues and affect systemic metabolism by interacting with central nervous system (Burýsek and Houstek, 1997; Hondares et al., 2011; Whittle et al., 2012; Wang et al., 2014). Peptidase M20 domain containing 1 (PM20D1) and Slit2 are two newly identified batokines that improves glucose homeostasis as well as regulate thermogenesis which might be used for the treatment of obesity and obesity associated metabolic disorders (Long et al., 2016; Svensson et al., 2016). In summary, adipokine/batokine-centered therapeutic strategies could pave the way for treatment of metabolic diseases and cancers.

## Inflammatory Mediators in Obesity

Development of chronic low grade systemic inflammation is one of the primary consequences of obesity (Bekkering et al., 2020). High fat diet induces the expression of pro-inflammatory cytokines and inflammatory responsive proteins in the hypothalamus (an important part of brain responsible for controlling hunger and thermogenesis). Leptin and insulin provide signals to specific neurons in the hypothalamus to report about the energy stocks in response to high fat diet. This signaling is accompanied by an increased expression of c-Jun N-terminal kinase (JNK) and nuclear factor-κB (NF-κB) and thereby inducing IR in the hypothalamus (De Souza et al., 2005). Moreover, depletion of medio-basal hypothalamus (MBH) in mice resulted in enhanced leptin signaling and reduced food intake, signifying the importance of inflammation in hypothalamus-related weight gain (Valdearcos et al., 2014). In addition to this, consumption of HFD is accompanied by unfavorable changes in gut microbiota (a decrease in ratio of Firmicutes to Bacteroidetes), metabolic profile of feces and plasma proinflammatory factors (PGE_2_ and TXB_2_) which adversely affect the health of young adults (Wan et al., 2019).

**FIGURE 3:**
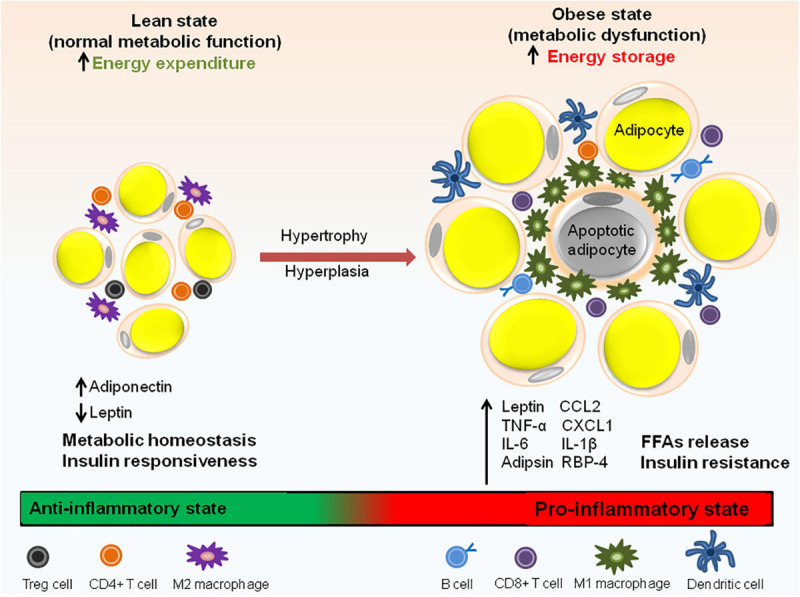
Immune cell distribution in lean and obese state. In lean adipose tissue with normal metabolic function, M2 macrophages are uniformly distributed throughout the tissue. The lean AT milieu also consists of CD4+ T cells and Treg cells having anti-inflammatory properties. Adiponectin to leptin ratio is high which contributes to the insulin responsive state of the adipocytes. In obese state, macrophages switch to M1 type which forms a crown like structure (CLS) around the adipocytes. Adipocyte hypertrophy results in the rupture of adipocytes and releases FFAs. In addition to M1 macrophages, obese state is also associated with an increase in CD8^+^ T cells, dendritic cells and IgG antibody producing B cells responsible for pathogenic state of AT. Obesity is also associated with abnormal adipokine profile, i.e., increased release pro-inflammatory adipokines. Aberrant secretion of adipokines (leptin, IL-6, adipsin, RBP-4, and IL-1β), chemokines (CCL2 and CXCL1) and macrophage factors (TNF-α) causes metabolic dysfunction and insulin resistance.

In healthy and lean individuals, the resident immune cells of adipose tissue are indispensable for its function but in individuals with obesity, inflammation of adipose tissue is one of the major contributors to metabolic dysfunction including systemic IR and/or glucose intolerance. The resident cells of both innate and adaptive immune system in the adipose tissue take part in this process (Figure 3) and are described below.

### Innate Immunity

Initial evidence to understand the connection between obesity and inflammation came from the finding that IR in the adipocytes is induced by macrophages (Pekala et al., 1983). Later, tumor necrosis factor-α (TNF-α) was identified as the molecule which mediated obesity-linked IR (Hotamisligil et al., 1993). Adipose tissue macrophages (ATMs) from lean mice show a different profile than ATMs of mice with obese phenotype. DIO shifts the activation state of ATMs from M2 anti-inflammatory state to M1 pro-inflammatory state exemplified by an increased expression of genes encoding TNF-α and NOS-2, which contribute to pathophysiological repercussions of obesity (Lumeng et al., 2007). Studies suggest that MCP-1/CCR2 axis is responsible for adipose tissue inflammation and development of obesity and IR (Kanda, 2006; Weisberg et al., 2006). Factors secreted from ATMs blocks the insulin action in adipocytes by down-regulating IRS-1 and GLUT4. Additionally, TNF-α neutralizing antibodies could partially reverse the IR induced by macrophage- conditioned media *in vitro* (Lumeng et al., 2007). ATMs isolated from mice and humans with obese phenotype have markers for increased *de novo* synthesis of phosphotidylcholine (PC) biosynthesis. Deletion of phosphocholine cytidylyltransferase A (a rate-limiting enzyme in *de novo* PC synthesis) in a macrophage-specific manner improved adipose tissue inflammation and IR (Petkevicius et al., 2019). Additionally, Galectin-3 (Gal-3), a lectin secreted by macrophages, has been found to directly bind to insulin receptor and inhibit the downstream insulin signaling. Gal-3 could be used as an important target for treatment of IR as its inhibition in mice improved insulin sensitivity and glucose tolerance (Li P. et al., 2016). Latest studies have also started to identify epigenomic alterations in macrophages that determine their sensitivity upon metabolic stress induced by obesity. A co-repressor complex containing G protein pathway suppressor 2 (GPS2) was identified as one such epigenomic modifier whose function and expression in macrophages is dependent on the disease state (Fan et al., 2016). Moreover, activation of inflammasome (a protein complex facilitating maturation of pro-inflammatory cytokines IL-1β and 1L-18 by caspase-1 mediated cleavage) is crucial for impairment of insulin signaling in target tissues. It is observed that the presence of free fatty acids in HFD triggers the activation of NLRP3-ASC inflammasome in macrophages by AMPK autophagy-ROS signaling pathway resulting in impaired insulin signaling (Wen et al., 2011). The role of melatonin in alleviating inflammasome-induced pyroptosis by blocking NF-κB/gasdermin D (GSDMD) signal in adipose tissue of mice has also been observed (Liu et al., 2017). Receptor for advanced glycation end products (RAGE), which is highly expressed in monocytes and macrophages and its ligand, high mobility group box 1 (HMGB1), are also found to be associated with development of obesity. Blockage of RAGE or neutralization of HMGB1 prevented HFD induced weight gain and improved glucose tolerance in mice model (Song et al., 2014; Montes et al., 2015). Also, the depletion of visceral adipose tissue macrophage from mice downregulated the genes involved in gluconeogenesis and lipogenesis which conferred protection from HFD induced obesity, IR and hepatic steatosis (Bu et al., 2013).

HFD is also reported to change the gut microbiome and cause dysbiosis which is considered one of the main factors contributing to colorectal cancer (CRC) susceptibility. Activation of MCP-1/CCR2 axis mediated by HFD-induced dysbiosis accelerated the incidences of advanced colorectal neoplasia (Liu et al., 2020). Specific gut bacteria also serve as a source of lipopolysaccharide (LPS) and increase the intestinal permeability along with the increase in systemic concentration of TNF-α and IL-6 in patients with T2D (Jayashree et al., 2014). A recent study demonstrated the role of TLR4 in LPS and saturated fatty acid mediated adipocytes dysfunction by stimulating inflammatory changes in adipocytes and macrophages (McKernan et al., 2020).

In addition to macrophages, other cells of innate immune system such as dendritic cells, mast cells, and neutrophils also contribute to development of obesity and IR. Accumulation of plasmacytoid DCs (pDCs) during obesity induces AT inflammation and T2D through their IFN-producing ability. IFNAR^–/–^ mice and the mice lacking pDCs failed to develop obesity and other metabolic complication upon feeding with HFD (Hannibal et al., 2017). Recently, a gene ontology (GO) analysis identified the association of obesity with increased percentage and gene activation of neutrophils in young African-American male population (Xu et al., 2015). Additionally, genetic deficiency or pharmacological stabilization of mast cells was found to ameliorate glucose homeostasis as well as weight gain due to obesity (Liu et al., 2009). However, a novel study in human subjects identified the role of mast cells in cold-induced subcutaneous WAT beiging independent of BMI. This adipose beiging was attributed to release of histamine during mast cell degranulation (Finlin et al., 2019).

In total, macrophages along with other cells of innate immune system contribute in the development of obesity and insulin resistance.

### Adaptive Immunity

While most of the studies on obesity, inflammation and IR are majorly directed toward the role of macrophages, recent investigations points to the significant participation of adaptive immune system in regulating obesity associated metabolic anomalies (Nishimura et al., 2009). A study reported that in obese state, CD8^+^ T cells helped in macrophage recruitment and caused adipose tissue inflammation. Moreover, genetic or immunological depletion of CD8^+^ T cells lowered the macrophage infiltration and adipose tissue inflammation, thereby ameliorated systemic IR. On the contrary, adoptive transfer of CD8^+^ T cells to CD8-deficient mice exacerbated AT inflammation (Nishimura et al., 2009). Mice with obese phenotype and lacking αβ T cell (TCRb−/− mice) exhibited reduced inflammation of adipose tissue and skeletal muscle suggesting the important role of T_*H*_1 cells in regulating inflammation and IR in obesity (Khan et al., 2014). Recently, a unique population of regulatory T cells, i.e., CD4^+^Foxp3^+^ T_*reg*_ cells, having anti-inflammatory properties were found to be highly enriched in visceral fat of mice with lean phenotype (Feuerer et al., 2009) and PPARγ was the central molecular initiator for accumulation and functioning of T_*reg*_ cells (Cipolletta et al., 2012). In addition to T cells, B cells also promote IR through activation of proinflammatory macrophages, T cells and production of pathogenic IgG antibodies. Depletion of B cells using anti-CD20 mAb in early stage of the disease can have therapeutic benefits in managing IR and associated co-morbidities (Winer et al., 2011). Also, B-cell null mice were found to be protected from obesity and systemic inflammation and had an increased ratio of anti-inflammatory regulatory T cells (DeFuria et al., 2013).

Altogether, these studies highlight the importance of both arms of immune system in adipose tissue inflammation and systemic IR in obese condition. Understanding the relationship between adipose tissue and immune cells could provide therapeutic targets for treating obesity and IR in future.

## Transcriptional Regulation of Adipocyte Differentiation

**FIGURE 4:**
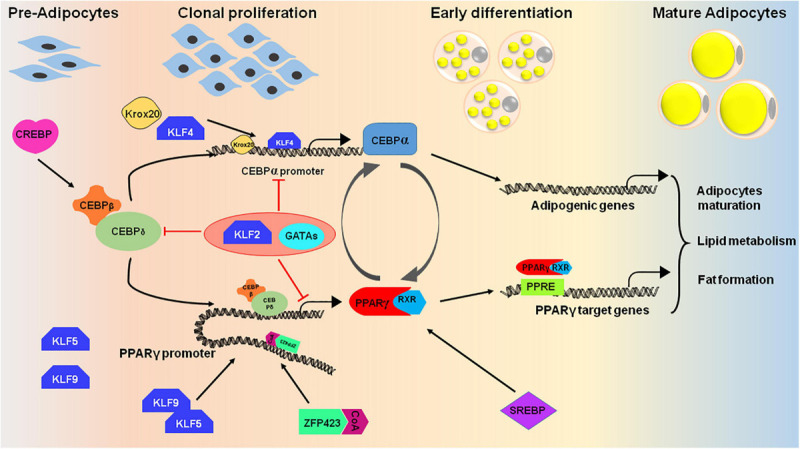
Cascade of transcription factors in adipocyte differentiation. Many transcription factors act as positive regulators that express at different stages of adipocyte differentiation (pre-adipocytes to mature adipocytes). The differentiation process initiates upon induction of cells with adipogenic cocktail which helps in activation of certain transcription factors like CREBP, KLF4, 5, and 9, and CEBPβ/δ. CEBPβ and CEBPδ triggers the second wave of adipogenesis by activating PPARγ, CEBPα and SREBP. The PPAR proteins dimerizes with retinoic X receptor (RXR) for interaction with target promoters containing PPAR-response elements. PPARγ and CEBPα are the key proteins that targets the essential genes required for adipogenesis. To regulate the process of adipogenesis, some transcription factors like KLF2 and GATA2/3 act as negative regulators and inhibits the expression of CEBPα and PPARγ by direct or indirect repression of the transcription cascade.

Multistage differentiation of pre-adipocytes or mesenchymal stem cells to adipocytes involves numerous transcription factors. The expression of these wide ranges of transcription factors regulates the differentiation process either positively or negatively. The core factors, PPARγ and C/EBP-α, along with several other proteins regulate the expansion of pre-adipocytes and thereby formation of lipid droplets in mature adipocytes (Figure 4; Herrera et al., 1989; Wu et al., 1999; Birsoy et al., 2008).

### Positive Regulators of Adipogenesis

#### PPARγ and C/EBPs

PPARγ and C/EBPα are considered as the key regulators of adipogenesis that are vital for adipocyte differentiation both *in vitro* and *in vivo* (Rosen et al., 1999; Linhart et al., 2001). The initial stages of adipocyte differentiation require C/EBPβ and C/EBPδ that triggers the mitotic cell division and clonal expansion (Tang et al., 2003). During the cell cycle progression from G1 to S phase, C/EBPβ is hyper-phosphorylated which leads to the activation GSK-3β and MAPK followed by mitotic division (Tang et al., 2005). Activation of GSK-3β and MAPK induces the transcription of PPARγ and C/EBPα for terminal differentiation of adipocytes. Although C/EBPα is an essential factor for adipocyte differentiation but it requires the presence of PPARγ to establish the adipogenic phenotype. In PPARγ^–/–^ fibroblasts, C/EBPα was unable to induce any lipid accumulation whereas PPARγ could induce adipogenesis in C/EBPα^–/–^ fibroblasts (Rosen et al., 2002). However, the complexity of adipogenesis *in vivo* is quite different and is temporally regulated. While C/EBPα is important for all white adipogenic requirement of an adult, the terminal adipogenesis in an embryo is completely independent of C/EBPα but requires PPARγ (Wang Q.A. et al., 2015). Although important, the presence of C/EBPα is not essential for adipocytes survival in adult stage (Wang Q.A. et al., 2015). The significance of PPARγ was also observed in *Pparg* null mice wherein these mice eventually developed diabetic nephropathy (Toffoli et al., 2017). The loss-of-function mutations in human *PPARG* results in the development of familial partial lipodystrophy type 3 (FPLD3) and other serious metabolic anomalies. Recently, a study reported that patients with FPLD3, harboring Arg308Pro (R308P) and Ala261Glu (A261E) PPARγ variants responded satisfactorily to synthetic PPARγ agonists (Agostini et al., 2018). Additionally, the systemic deletion of PPARγ in mice caused total lipoatrophy accompanied by organomegaly and hypermetabolism. *Pparg*^Δ^^/^*^Δ^* mice also developed severe T2D and showed metabolic inflexibility (Gilardi et al., 2019). Altogether, the experimental data from different studies suggest that PPARγ is the master regulator of adipogenesis and the main role of C/EBPα is to maintain the expression of PPARγ.

#### Zinc Finger Proteins (ZFPs)

The family of ZFPs is known to regulate various biological functions and some of the ZFPs that play significant role in adipocyte differentiation are also well elucidated. Adipogenic stimulus results in increased expression of ZFP423 at both transcript and protein level in 3T3-L1 cells. Over expression of ZFP423 in non adipogenic cell line (NIH-3T3) resulted in their adipogenic differentiation via robust activation of PPARγ (Gupta et al., 2010). Furthermore, the overexpression of ZFP423 in low adipogenic cells resulted in increased competence of the cells to differentiate into mature adipocytes. However, the knockdown of ZFP423 in high adipogenic cells prevented their adipogenic differentiation. This differential regulation of ZFP423 in low and high adipogenic cells was found to be associated with DNA methylation of its promoter (Huang et al., 2012). Moreover, the recruitment of ZFP30 and its co-activator KRAB-associated protein 1 (KAP1) on PPARγ2 enhancer activates its expression and thus promotes adipogenesis (Chen et al., 2019).

Many other transcription factors like Sterol regulatory element-binding protein 1 (SREBP1), Cyclic AMP Response Element-Binding Protein (CREBP) and several proteins from Kruppel-like factor family (KLFs) like KLF4, KLF5, KLF9, KLF15 positively regulate the adipocyte differentiation at various stages by binding to the promoter of either PPARγ or C/EBPs (Tontonoz et al., 1993; Zhang et al., 2004; Oishi et al., 2005; Birsoy et al., 2008). For example, overexpression of SREBP1 in adipocytes as well as in HepG2 cells can induce PPARγ transcript expression suggesting that SREBP1 enhances PPARγ expression (Fajas et al., 1999). Likewise, CREBP positively regulate the expression of C/EBPβ by interacting with its promoter (Zhang et al., 2004). While KLF5 and KLF9 are known to bind to PPARγ2 promoter, KLF4 binds to C/EBPβ promoter along with Krox20, thereby regulating its expression in early phase of adipogenesis (Oishi et al., 2005; Pei et al., 2011).

### Negative Regulators of Adipogenesis

Various signaling pathways and transcription factors help in maintaining the expression of positive regulators. The intricate balance between the positive and negative regulators is required for the efficient and regulated conversion of pre-adipocytes to lipid loaded mature adipocytes. The absence of negative regulators or increased expression of positive regulators may result in obesity and related disorders. These transcription factors are potential targets to control obesity and metabolic disorders.

#### GATA-Binding Factors

These zinc finger proteins bind to various promoters to regulate the cellular development and differentiation. GATA2 and GATA3 are abundantly expressed in pre-adipocytes and their expression decreases during adipocyte differentiation (Tong et al., 2000). Constitutive expression of GATA2 and GATA3 results in their interaction with either C/EBPα or C/EBPβ thereby inhibiting their activity (Tong et al., 2005). In general, GATAs subdues the adipogenesis process by two pathways, i.e., by interaction with PPARγ promoter and by protein-protein interaction which hinders the expression of C/EBP protein (Tong et al., 2000, 2005). GATA protein works along with cofactor Friend of GATA (FOG) and C-terminal binding proteins (CTBPs). FOG and CTBP protein interact with GATA2 in pre-adipocytes and inhibits the terminal differentiation of adipocytes (Jack and Crossley, 2010). Downregulation of GATA2 led to the pathogenesis of diseases like aplastic anemia, which was reported to have elevated expression of PPARγ (Xu et al., 2009). A recent study described GATA3 as a target gene of KLF-7 which inhibits chicken adipogenesis (Sun et al., 2020). Altogether, the interaction of GATAs with numerous proteins at different stages of adipogenesis keeps the positive adipogenic regulators in check and maintains the metabolic homeostasis.

In addition to GATA- binding factors, several other proteins like Pref-1, SIRT1, HDAC9 and transcriptional modulator TAZ also negatively regulate the differentiation of adipocytes by inhibiting the positive regulators at different stages of adipogenesis (Moon et al., 2002; Kurtev et al., 2004; Hong et al., 2005; Chatterjee et al., 2011). In contrast to other KLFs, KLF-2 inhibits the differentiation of adipocytes by interaction with a consensus motif 5′-CNCCC-3′ present in PPARγ2 promoter, thus limiting its expression (Sen Banerjee et al., 2003).

## Epigenetic Regulation of Adipogenesis

**FIGURE 5:**
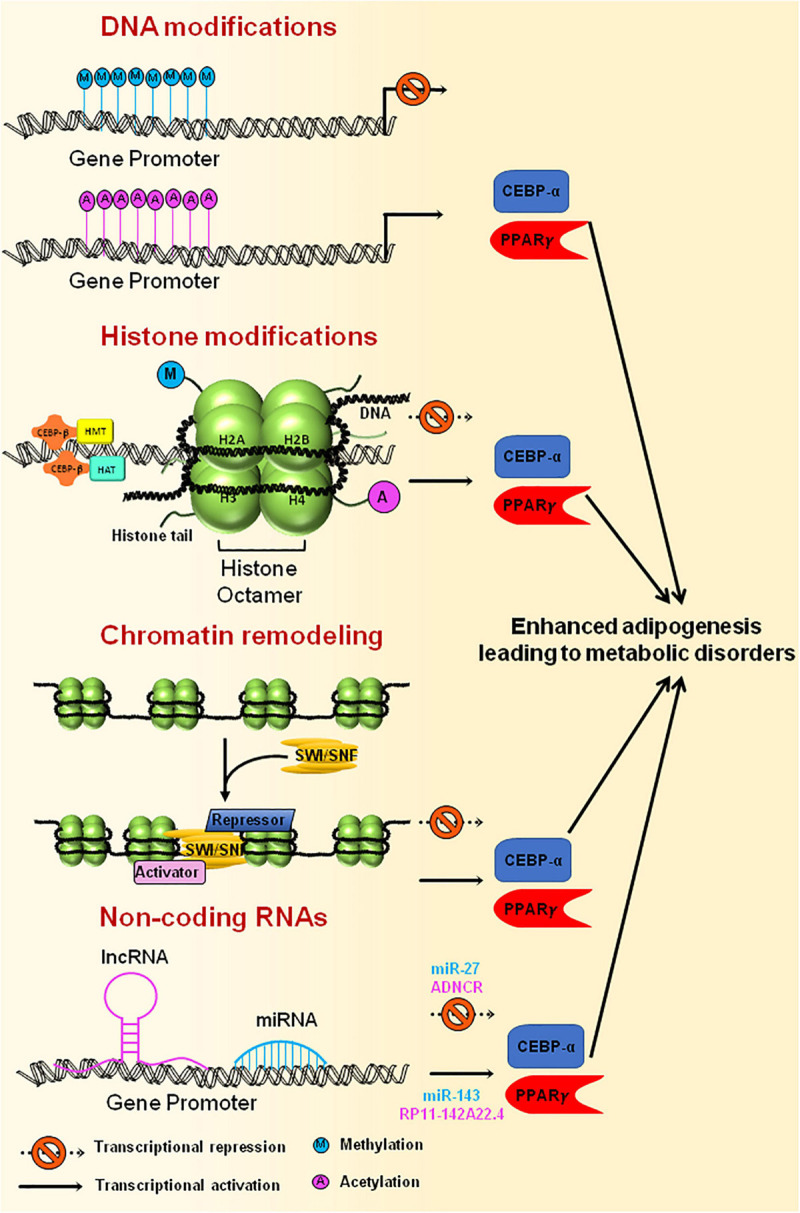
Epigenetic modification of genes involved in adipogenesis. Methylation of gene promoters, that are necessary for adipogenesis, results in inactivation of the genes leading to reduced adipogenesis, whereas acetylation of promoter region brings about active adipocyte differentiation. Histone modification through HATs or HMTs that are recruited at the gene promoter by CEBPβ results in either activation or repression of the genes that are essential for adipogenesis. Chromatin remodeling complexes, such as SWI/SNF, tends to change the chromatin structure, thereby making the DNA either accessible or inaccessible for the transcription of adipogenesis specific genes to happen. Non-coding RNAs also govern the transcription of master regulators of adipogenesis by activating (miR-143, RP11-142A22.4) or repressing (miR-27, ADNCR) the transcription of key genes required for adipogenesis. Uncontrolled expression of genes involved in adipogenesis could ultimately lead to metabolic disorders.

There are numerous epigenetic events involved at specific stages of adipocyte differentiation that eventually decide the fate of adipogenesis. Post Translational Modifications (PTMs) of histones such as Histone acetyltransferases (HATs), Histone deacetylases (HDACs), Histone methyltransferases (HMTs), and Histone demethylases (HDMs) have been reported to be crucial in shaping the adipogenesis process (Lizcano et al., 2011; Okuno et al., 2013). Along with histone PTMs, DNA methylation, chromatin remodeling and several microRNAs (miRNAs) also guide the adipogenesis program (Figure 5; Salma et al., 2004; Sakamoto et al., 2008; Peng et al., 2013). This part of the review focuses on the role of epigenetic regulation that ultimately dictates the adipocyte differentiation in normal scenarios and during metabolic disorders.

### Histone Modifications

The constantly varying histone modifications are responsible for controlling the expression of various regulators of adipogenesis process namely Pref-1, C/EBP(α/β), PPARγ2 and aP2 (Zhang et al., 2012). MLL3/MLL4 are the most important H3K4 methyltransferases that are known to prime the enhancer before their activation thereby determining the ultimate cell fate (Wang et al., 2016). MLL3/MLL4 and CBP/p300 are also known as the super enhancer epigenomic regulators and activators that control the chromatin landscaping during adipocyte differentiation (Lai et al., 2017). Additionally, the mutations associated with MLL2 are responsible for lowering glucose tolerance in mice that could result in T2D (Goldsworthy et al., 2013). Similarly, cases of congenital hyperinsulinemia were observed in human infants having mutated *MLL2* gene (Yap et al., 2019). A genome wide histone modification examination uncovered the histone modification pattern of H3 that is frequently associated with obesity and diabetes (Jufvas et al., 2013). A study conducted on hyperphagic (ob/ob) mice and in mice with DIO has shown an increase in the acetylation level of lysine (K9, K18) on histone H3 at the gene promoter of TNF α and CCL2 in the liver tissue (Mikula et al., 2014). Moreover, a decreased methylation pattern of histone H3 (H3K4me3) was observed under high Isocitrate Dehydrogenase 1– α-Ketoglutarate (IDH1–α-KG) conditions, thus regulating the brown adipocyte differentiation in mice (Kang et al., 2020). This could be used as a therapeutic target for various metabolic syndromes. In a recent experiment conducted on human VAT, enhanced H3K4me3 marks were observed on the promoter region of various genes that are involved in adipogenesis, lipid metabolism and inflammatory pathways (Castellano-Castillo et al., 2019a). There are various protein arginine methyltransferases (Prmts) that are involved in regulating the expression of numerous regulators of adipogenesis. Studies revealed that overexpression of Prmt5 eventually promotes adipocyte differentiation by upregulating PPARγ2 gene expression via forming an immature Promoter-enhancer looping (LeBlanc et al., 2012; Leblanc et al., 2016). Whereas, Prmt6 acts as a negative regulator of adipogenesis and is known to repress the activity of PPARγ (Hwang et al., 2019). Unlike Prmt5 and Prmt6, knockdown and overexpression of Pmrt7 did not affect the adipocyte differentiation; hence not all Prmts are important for regulating adipogenesis (Imbalzano et al., 2013).

Differential expression of HDACs is known to be associated with various metabolic conditions. For example, a case control experiment conducted on women with normal weight and women with obesity, showed a differential expression of HDAC2/4/5/6 that could be associated with obesity and inflammatory reactions related to obesity (Shanaki et al., 2020). Also, mice lacking *Hdac9* or *Hdac11* gene were found to have an increased whole-body energy consumption which protected them against DIO (Chatterjee et al., 2014; Sun et al., 2018). Moreover, the alteration in class I HDAC activity has been shown to shift the white adipocytes phenotype toward brown-like phenotype by modifying the histone marks (Ferrari et al., 2020). In addition to other HDACs, Class III HDACs (Sirtuins) are also known to regulate the adipocyte differentiation. Studies involving HFD*-*fed, *Sirt1* knockout mice model showed an increase in adipose tissue mass by promoting PPARγ activity, indicating a negative correlation between Sirt1 and adipogenesis (Mayoral et al., 2015). Complete *Sirt7* knockout in mice resulted in reduction of white adipose tissue which indicates that Sirt7 is a positive regulator of adipocyte differentiation (Fang et al., 2017). Additionally, mutation in Sirt6 has been found to disturb the adipogenesis phenomenon, as Sirt6 is essential for regulating the mitotic clonal expansion in cells via suppressing the expression of Kinesis heavy chain isoform 5C (Chen et al., 2017). A study in human SAT and VAT has shown that reduced Sirt1 and Sirt2 expression was associated with increased visceral adipose stem cells differentiation ability (Perrini et al., 2020). Similarly, knockdown of Jumonji domain containing protein 6 (JMJD6), a histone arginine demethylase, results in reduced expression of PPARγ2 and C/EBPα both at transcript as well as post transcription level, thereby inhibiting adipocyte differentiation (Hu et al., 2015). Later, studies revealed that the positive regulation of adipogenesis by JMJD6 is independent of its catalytic domain and requires its AT-hook like domain to interact with other important adipogenesis regulators by acting as a scaffold protein for them (Reyes-Gutierrez et al., 2019).

Statins are DNA methylation inhibitors and are known to regulate blood cholesterol but recently they are also found to be associated with a high risk of causing T2D. Statin treatment tends to reduce the methylation pattern on HDAC9 promoter that results in a reduced expression of key regulators of adipogenesis (Khamis et al., 2020). Keeping in consideration all the information obtained from various studies, modification of histone marks appears to be a potential therapeutic target for addressing numerous metabolic disorders.

### DNA Methylation

The DNA methyltransferase (DNMT) family comprises of five main enzymes which regulate the *de novo* DNA methylation (DNMT3A/3B) and adds methylation marks during replication (DNMT1) (Weber et al., 2007; Lyko, 2018). Earlier it was reported that reduced expression of DNMT1 by a novel miRNA (ACL-miR-148a) in 3T3-L1 cell line resulted in the promotion of adipogenesis by decreasing the DNA methylation marks on *PPARγ* (Londono Gentile et al., 2013). Later, it was revealed that DNA methylation has a biphasic effect on adipogenesis process where in the early stage, inhibition of methylation by 5-aza-dC promoted the adipocyte differentiation, while in late stage it inhibited the adipogenesis (Yang et al., 2016). Additionally, an altered global DNA methylation pattern during metabolic disorders has been observed on various genes involved in adipocyte differentiation, lipid metabolism, and inflammation (Castellano-Castillo et al., 2019b). A novel methylase enzyme, METTL4, responsible for the methylation of N^6^- methyladenine (6ma) was found to promote adipogenesis in 3T3-L1 cells (Zhang Z. et al., 2020).

Modified cytosine(C) residue, 5-methylcytosine (5mC), established by DNMTs could easily be reverted to unmodified C by ten eleven translocation (TET) enzymes (Wu and Zhang, 2017). The mouse model having TET1/2 double knockout was found to have developmental abnormalities along with adipocyte differentiation defects because of the associated epigenetic instabilities (Wiehle et al., 2016). Recently global levels of 5mC were examined in the genome of 3T3-L1 cells. Among all the DNA demethylases, TET2 exhibited the major effect on adipocyte differentiation studies. Knockdown of *Tet2* resulted in enhanced adipogenesis and hence it is considered as an anti-adipogenic demethylase (Hou et al., 2020). Still a detailed gene knockout study in mice model is needed to further determine the involvement of TET1/2 in regulating adipogenesis.

### Chromatin Dynamics and Remodeling

A dynamic chromatin is indispensable for an effective replication and transcription process to take place. Various ATP-dependent remodeling factors are required to carry out the chromatin remodeling. The SWItch/Sucrose Non Fermentable (SWI/SNF) is one such ATP-dependent family of chromatin remodeling complex which by utilizing brahma (BRM) or brahma-related bromodomain protein (BRG) makes the chromatin access easy through the rearrangement of nucleosomes (Kadoch and Crabtree, 2015). A study showed that involvement of C/EBP is essential in recruiting the SWI/SNF enzymes on PPARγ2 promoter in order to proceed with the adipogenesis process (Salma et al., 2004). Another study demonstrated that C/EBPα transactivation element III (TE-III) interacts with SWI/SNF chromatin remodeling complex to collaborate with TBP/TFIIB for adipocyte differentiation (Pedersen et al., 2001). Knockdown of Prmt5 has been found to decrease the binding of BRG1, a SWI/SNF ATPase that is required for activating PPARγ2. It eventually resulted in reduced adipogenesis because BRG1 failed to interact effectively with the PPARγ2 chromatin locus in the absence of Prmt5 (Leblanc et al., 2016). Although, the role of SWI/SNF for the activation of enhancers during cancer development has been widely studied (Nakayama et al., 2017), but its involvement in activating adipogenesis related enhancers for effective gene expression is yet to be explored.

### Non-coding RNAs

There are several non-coding (nc) RNAs, small nuclear RNAs (snRNAs), microRNAs (miRNAs), and long nc RNAs (lncRNAs) that are extensively involved in regulating various essential genes or transcription factors involved in numerous biological processes (Mercer et al., 2009; O’Brien et al., 2018). Many of these miRNAs and lncRNAs are also known to control the adipogenesis process by regulating the expression transcription factors involved in adipocyte differentiation during normal and diseased conditions (Hilton et al., 2013; Arner and Kulyté, 2015; Chen et al., 2018).

Initial miRNA microarray studies highlighted the increased expression of miR-143 in preadipocytes where it promoted the adipocyte differentiation via controlling the levels of ERK5 protein (Esau et al., 2004). An intronic miRNA, miR-33, which is present within the SREBP-2 gene has come up as an essential non coding RNA which transcriptionally controls the cholesterol homeostasis by inhibiting the adenosine triphosphate–binding cassette (ABC) transporter (Najafi-Shoushtari et al., 2010; Rayner et al., 2010). Further study conducted on miR-33 knockout mice has revealed an enhanced expression of SREBP-1 in these mice which leads to obesity and various hepatic complications (Horie et al., 2013). Additionally, miR-27 as well as miR-130 gene family were found to inhibit the master regulators (PPARγ, C/EBPα) of adipocyte differentiation and therefore considered negative regulators of adipogenesis (Lin et al., 2009; Lee et al., 2011). A microarray study has shown the presence of PPARγ regulated differential miRNA expression profile in human subcutaneous and visceral fat tissues. An increase in the expression of miR-378 has been observed upon pioglitazone (PPARγ agonist) treatment, where it was found to enhance the adipocyte differentiation in the subcutaneous tissue but no effect was seen on visceral tissue (Yu et al., 2014). miR-146 and miR-93 were found to inhibit the expression of Sirtuins (Sirt1, Sirt7, respectively) in order to regulate adipogenesis (Ahn et al., 2013; Cioffi et al., 2015). Another mi-RNA that came up as a positive regulator of adipocyte differentiation is miR-125-5p. It has been found to suppress the genes involved in cell cycle progression (G1/S) and results in enhanced expression of key adipogenesis associated genes (Ouyang et al., 2015). Recent transcriptome analysis performed on human mesenchymal stem cells focused upon those miRNAs that are somehow involved in the lipid droplet formation during adipogenesis and could be used as disease biomarkers for various metabolic disorders (Yi et al., 2020). A miRNA originated from hepatic exosome, miR-130a-3p, was found responsible for mediating a tissue cross-talk in order to regulate the glucose intolerance by inhibiting the PH domain leucine -rich repeat protein phosphatase 2 (PHLPP2) during adipocyte differentiation (Wu et al., 2020). Also, the novel role of miR-196b-5p in promoting adipogenesis by inhibiting the expression of tuberous sclerosis 1 (Tsc1) and transforming growth factor-β receptor 1 (TGFBR1) was established (Shi et al., 2020).

A few circulating lncRNAs, despite having no functional outcome, have also been found to be differentially expressed among lean people and in people with obesity. A transcriptome study carried out in bovine preadipocytes found a lncRNA, adipocyte differentiation-associated long non-coding RNA (ADNCR), that suppressed adipogenesis by inhibiting miR-204 which is a known repressor of Sirt1 (Li M. et al., 2016). Also, silencing of lncRNA H19 in BAT reduced adipocyte differentiation, whereas its absence enhanced adipogenesis in WAT (Schmidt et al., 2018). A global expression pattern study resulted in the identification of RP11-142A22.4, expression of which was found to be highly increased during adipocyte differentiation and hence it could be used as a therapeutic target for obesity (Zhang T. et al., 2020). Despite of all the available literature, a lot is yet to be explored in order to implement the findings for the treatment of metabolic disorders.

## Environment-Epigenetic Interaction

The prevalence of obesity in modern environment can be understood with regard to evolution (Lev-Ran, 2001). Our primeval ancestors favored “thrifty” genotype that enabled them to efficiently store fat during a period of famine. The hunter gatherers had the cycles of feast and famine interspersed with cycles of physical activity and rest. Their ability to conserve energy by storing fat provided them with genetic advantage for selecting this genotype for unfavorable conditions (food scarcity). Therefore, these individuals were more likely to survive the periods of famine than lean individuals who were more prone to infectious diseases (Eaton et al., 1988; Chakravarthy and Booth, 2004). We, the modern day humans, have the continuous supply of food and are relatively physically inactive which abrogates the evolutionary programmed feast-famine and physical activity-rest cycles. So, carrying the thrifty genotype, turned out to be a risk factor for developing obesity and metabolic diseases (Chakravarthy and Booth, 2004).

**FIGURE 6:**
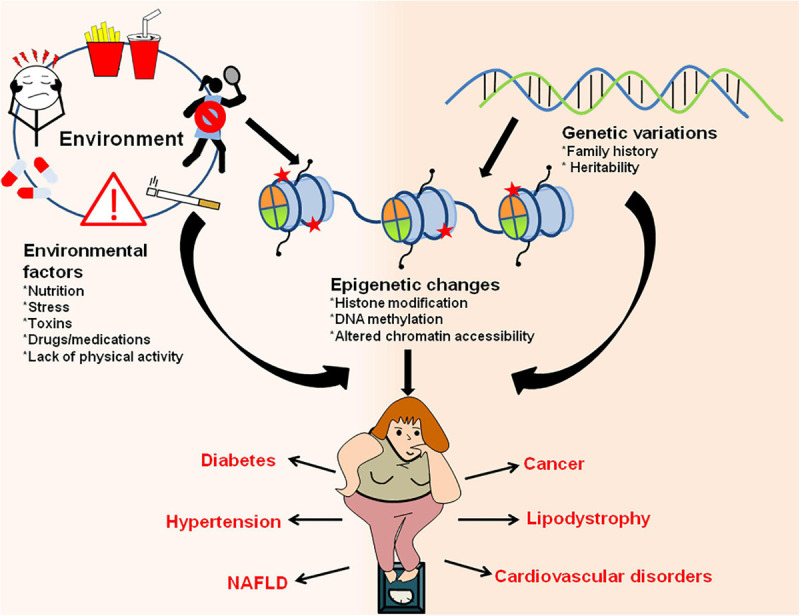
Interaction between environment/genetic factors and epigenetic changes in establishment of obesity and obesity-associated metabolic disorders. Environmental factors like exposure to drugs/toxic chemicals, lack of physical activity, sedentary lifestyle, poor and unhealthy diet, stress/anxiety, smoking/alcohol abuse along with genetic makeup of an organism can have direct influence on epigenetic marks and result in increased adiposity. The changes in epigenetic landscape through various histone modifications, changes in chromatin accessibility and DNA methylation results in obesity and other metabolic disorders like diabetes, hypertension, lipodystrophy, cardiovascular diseases, NAFLD, cancer, etc.

The pathophysiology of obesity is highly complex and involves the interplay of environmental factors, lifestyle changes (nourishment, exercise, exposure to noxious substances) and gene expression factors. Additionally, the gene expression changes are believed to have associated epigenetic changes that link epigenetics with obesity (Figure 6; Youngson and Morris, 2013; Albuquerque et al., 2017). Several medications and environmental toxins are known to induce adiposity. For example administration of valproic acid (VPA; a histone deacetylase) in children for treatment of epilepsy lead to an increased risk of developing metabolic and endocrine disorders (Carmona-Vazquez et al., 2015). Sodium VPA is also linked with an increase in BMI, increased leptin levels, IR and hyperinsulinemia in these children (Rehman et al., 2017).

Nutrition and the type of diet directly influence epigenetic marking and have a role to play in obesity and related metabolic disorders. DNA and histone methyltransferases uses S-adenosyl-methionine (SAM) as methyl donors, availability of which is directly influenced by diet (Zeisel, 2009). SAM is formed by the diet supplemented with folate, Vitamin B6, B12, choline, and methionine and is critical for fetal development where it help in DNA methylation and proper brain development of the child. Deficiency of methyl donors might result in lifelong changes in gene expression and results in several health problems like IR and fatty liver (Sinclair et al., 2007). Moreover, supplementation of methyl donors can improve NAFLD in rats fed on obesogenic diet pointing to the fact that methyl supplementation might prove to be protective against obesity (Cordero et al., 2013). Some food components such as polyphenols and organosulfur compounds have also shown positive results in lowering obesity, inflammation, oxidative stress and cancers (Milagro et al., 2013). One such organosulfur compound is sulforaphane which is naturally present in cruciferous vegetables. Sulforaphane administered as broccoli extract reduced the fasting blood glucose and glycated hemoglobin (HbA1c) in patients with obesity and T2D (Axelsson et al., 2017).

Chemicals present in our environment, termed as obesogens, can also affect a person’s susceptibility to obesity by helping in adipocyte differentiation *in vitro* and storage of fat *in vivo* (Gru et al., 2006). One of the ubiquitous obesogen is organotin, like tributyltin (TBT) which is widely used in industries and agriculture. Human exposure to organotin is possible through consumption of seafood contaminated with TBT used in marine shipping applications (Mattos et al., 2017). TBT activates all three RXR–PPAR-α, -γ, -δ heterodimers, mainly through its interaction with RXR and thereby promotes adipogenesis and lipid accumulation (le Maire et al., 2009). Other obesogens include phthalates, persistent organic pollutants, components of plastics and epoxy resins. In addition to acting through nuclear receptors, these obesogens can also induce epigenetic changes and alters the chromatin accessibility or architecture in adipose tissue (Chamorro-Garcia et al., 2017). RXR activation also alters the expression of enhancer of zeste homolog 2 (EZH2) which results in genome-wide reduction and redistribution of histone 3 lysine 27 trimethylation (H3K27me^3^) repressive marks and promote adipose-lineage commitment (Shoucri et al., 2017).

Apart from the above listed factors, there are numerous other societal factors such as sleep patterns, sleep deprivation, chronic shift working which alter the circadian clock genes and disrupt metabolic integrity. Even a single night of sleeplessness can alter the transcriptional and epigenetic profile of circadian clock genes consequently resulting in reduced glucose tolerance and increased insulin sensitivity (Donga et al., 2010; Cedernaes et al., 2015; Morris et al., 2016).

## Transgenerational Inheritance of Obesity

Environmental stress/exposure can reprogram the epigenetic patterns of germ cells (egg and sperm) which associate with the development of altered phenotypes in future generations through epigenetic transgenerational inheritance (Anway et al., 2005; Skinner et al., 2013). As a result of early life developmental plasticity, the risk of obesity begins *in utero*. This idea is in accordance with the Developmental Origins of Health and Diseases (DOHaD) hypothesis which seeks to understand the relationship between perinatal environmental conditions and disease manifestation in adulthood (Barker et al., 1989; Ravelli et al., 1999). Adipose tissue is regarded as the main target of developmental programming in a sex- and depot-specific manner. Despite of the difference in developmental time windows, similar mechanisms of adipose tissue programming exist across species. Nutritional status of mother largely affects the reprogramming of offspring’s adipose tissue resulting in increased adipogenesis and lipogenesis, increased inflammation and impaired sympathetic activity thereby rendering them to disproportionate fat accumulation (Lecoutre et al., 2018). The excessive fat accumulation results in leptin and insulin resistance in these individuals predisposing them to metabolic syndrome (Muhlhausler and Smith, 2009). Maternal obesity in mice reduces the DNA methylation on Zfp423 promoter (i.e., reduced histone modification H3K27me3), which is correlated with enhanced Zfp423 expression and adipogenesis in fetal progenitor cells which thereby predisposes the offspring to obesity and metabolic dysfunction later in life (Yang et al., 2013). Gestational obesity (OB) in rats is responsible for broad changes in lipogenic and adipogenic genes in the WAT of offspring. OB-dam offsprings shows an increased mRNA expression of SREBP-1, GLUT4 and a greater AKT phosphorylation. They also exhibit increased expression of adipogenic regulators like PPARγ, C/EBP-α and C/EBP-β associated with differentiation of WAT stromal-vascular cells. These transcriptional changes are also associated with certain epigenetic changes like alteration in DNA methylation of CpG sites and CpG island (CGI) shores proximal to developmentally important factors including Zfp234 and C/EBP-β (Borengasser et al., 2013). Evidence suggests that the ratio of omega-6 (n-6) relative to omega-3 (n-3) polyunsaturated fatty acids (PUFA) is essential in regulating perinatal adipogenesis (Rudolph et al., 2018). A diet rich in n-3 PUFA decreased adipose tissue mass and prevented the development of obesity in rodents (Madsen et al., 2005). Moreover, offsprings of transgenic mothers with low n-6/n-3 PUFA ratio in plasma during gestation and lactation had smaller adipocytes, reduced gene expression of certain pro-adipogenic markers (Pparg2, Fabp4, and Plin1), elevated circulating levels of adiponectin and hypermethylated proximal promoter of Pparg2 (Rudolph et al., 2018). Exposure to HFD during pregnancy may affect glucose and lipid metabolism of female offsprings through epigenetic changes in *Leptin* (methylation of H4K20) and *Adiponectin* (decrease in acetyl H3K9 levels and increase in dimethyl H3K9 levels) genes for multiple generations. These epigenetic changes result in metabolic abnormalities like weight gain, glucose and insulin intolerance, hypertension, abnormal adipocytokine levels, etc. The effects are much stronger if the HFD *in utero* continues for multiple generations. However, a switch to normal diet *in utero* may prevent the epigenetic changes caused by HFD and eliminate the metabolic effects after the normal diet is restored for three generations (Masuyama et al., 2015). Not only maternal obesity but paternal obesity also contributes to metabolic disturbances in future generations. Diet induced paternal obesity modulates the sperm miRNA profile and methylation status of germ cell which initiate the transmission of obesity and metabolic diseases to future generations and adversely affect the health of offspring (Fullston et al., 2013).

Apart from the dietary factors, several other environmental insults that have been identified in recent times which induce transgenerational inheritance of obesity and related metabolic disorders are listed in Table 1.

**TABLE 1:** Environmental insults that can induce transgenerational inheritance of obesity and obesity associated metabolic disorders.

**Environmental factor**	**Category**	**Effects**	**References**
**4,4′-dichlorodiphenyltrichloroethane (DDT) 4,4′-dichlorodiphenyldichloroethylene (DDE) Methoxychlor**	Pesticide	Weight gain, altered glucose homeostasis; increased adipogenesis and lipid accumulation in 3T3-L1 cells; F3 generation sperm epimutations and differential DNA methylation regions (DMR). Increased adiposity and tumor development	Howell and Mangum, 2011; Skinner et al., 2013; Kim et al., 2016 Manikkam et al., 2014
**Bis-phenol-A (BPA) Phthalates**	Plastics	Increased adiposity and DNA methylation epimutations in sperm related to obesity genes	Manikkam et al., 2013
**Cadmium Lead**	Heavy metals	Increased risk of obesity in children. Increase in weight and triglycerides level, hepatic lipid accumulation, DNA hypermethylation	Green et al., 2018 Sun et al., 2017
**Tributyltin (TBT)**	Organotins	Increased WAT depot weights, hypertrophy, hyperplasia; hepatic lipid accumulation in three subsequent generations; transmissible changes in chromatin organization; global changes in DNA methylation; induce phenotype resembling NAFLD through atleast three subsequent generations.	Chamorro-García et al., 2013; Chamorro-Garcia et al., 2017
**Maternal HFD Paternal HFD**	Overnutrition	Weight gain, glucose intolerance, hypertension, abnormal adipocytokine levels, epigenetic changes in adipocytokines, leptin and adiponectin genes Changes in sperm miRNA profile and methylation status of germ cell, increased adiposity in future generation.	Masuyama et al., 2015 Fullston et al., 2013
**Paternal prediabetes**	Metabolic defect	Alteration in methylome pattern of sperm, transgenerational inheritance of diabetes (glucose intolerance and insulin resistance in offspring).	Wei et al., 2014

## Therapeutic Strategies for Obesity Treatment

Several clinical and epidemiological studies identify behavioral patterns including dietary habits as well as individual genetics to have direct correlation with metabolic syndrome and obesity. Apart from this, gut microbiome and environmental conditions also play a vital role in onset of obesity (Sonnenburg and Bäckhed, 2016). Additionally, if the calorie uptake is lowered, then the metabolic flux shifts toward catabolism of adipose tissues and glycogenolysis resulting in weight loss (Anton et al., 2018). Most of the strategies to control or treat obesity rely on calorie restriction. Drugs are designed either to lower the appetite for food or inhibit the absorption of tri-acyl glycerols. After several decades of research only a few drugs have been FDA approved for treatment of obesity and its associated disorders. Treatment of obesity is highly complex because most of the targets are either undruggable or have pronounced side effects due to their function in cellular homeostasis. Most of the available appetite-suppressant drugs act on the peripheral nervous system, targeting noradrenergic receptors resulting in reduced food intake by modulating the signaling of monoamine neurotransmitters such as serotonin and norepinephrine. Sibutramine (Meridia, Abbott), an appetite suppressant, first approved in November 1997 for the long-term treatment of obesity, showed some promising results. It works by inhibiting 5-HT and norepinephrine reuptake in the hypothalamus (Astrup et al., 1998). However, in the year 2010 the drug was withdrawn from the market due to increased cardiovascular complications (James et al., 2010). Fenfluramine is another drug which targets serotonergic 5-HT2 receptor agonist and σ1 receptor antagonist. Fenfluramine and dexfenfluramine were also withdrawn from United States in the year 1997 because of heart valve damage (Connolly et al., 1997; Smith et al., 2006). Phentermine is structurally similar to amphetamine which is prescribed for short term weight loss. It stimulates the central nervous system to release norepinephrine in the hypothalamus which increases the heart rate and blood pressure and decreases appetite (Rothman et al., 2001). The combination of phentermine with fenfluramine or dexfenfluramine was once used to treat obesity. Due to their side effects and contradictions such as dizziness, insomnia, dry mouth and cardiovascular problems, it is classified as schedule IV drug and could only be prescribed for short term usage. CB1 receptor, which is widely expressed in the central nervous system, is another target for treatment of obesity. Rimonabant, an inhibitor of CB1, increases adiponectin production in adipocytes leading to increased fatty acid oxidation (Pagotto et al., 2005). Rimonabant was approved in Europe in 2006, but it was withdrawn due to its adverse effect on patients such as anxiety, depression and suicidal tendencies in the clinical trials.

Apart from appetite suppression, other strategies were derived which inhibited nutrient uptake and assimilation through suppression of gastrointestinal lipase. Orlistat is an approved drug in the United States and Europe for long term obesity treatment, targeting triacylglycerol lipase thereby reducing dietary fat uptake and weight gain. Orlistat is a safe drug, but it does have some gastrointestinal side effects such as stomach pain and uncontrolled bowel movement (Ballinger, 2000). Topiramate is a sulfamate-substituted monosaccharide generally prescribed for migraine treatment. Topiramate works by inhibiting fructose 1,6-bisphosphatase, a rate limiting enzyme for gluconeogenesis, and controls blood glucose levels. Topiramate, however, has also been shown to suppress appetite and is found to be effective in weight reduction. Although, not FDA approved for the treatment of obesity, studies have demonstrated that it helps in weight reduction in individuals affected with obesity when administered in combination with phentermine (Colman et al., 2012; Cosentino et al., 2013).

Apart from classical therapeutic approaches, targeting the epigenetic regulators and factors governing adipogenesis is becoming a new hot spot for obesity treatment. The role of PPAR, an important component of adipogenesis and fatty acid oxidation, is investigated as a drug target for obesity. PPAR agonist bezafibrate showed efficacy in adipocyte dedifferentiation to preadipocytes by regulating the metabolic flux and β-oxidation (Cabrero et al., 2001; Vázquez et al., 2001). Also, treatment of another PPAR agonist GI259578A to AKR/J (AKR) mice resulted in increased mean size of WAT in the group of mice with obese phenotype as compared to the control group. Conversely, in mice with diabetic phenotype (*db/db)*, treatment of PPARγ agonist GW347845X resulted in 96.1% increased lipid storage in BAT and 15.4% decrease in WAT indicating a more complex mechanism of adipogenesis which needs to be understood before taking this drug to the clinics (Okamoto et al., 2007). Carnitine palmitoyltransferase 1 (CPT1) is another target for treatment of obesity as it helps in the entry of long-chain fatty acids into mitochondria for β-oxidation. Etomoxir, a CPT1 inhibitor, blocks the lipid transport thereby shifting metabolism toward glycolysis and oxidative phosphorylation (Schmidt-Schweda and Holubarsch, 2000).

Identification of blood-based epigenetic markers is emerging as a promising approach in early diagnosis of obesity and metabolic diseases. Such cell-free DNA (cfDNA)-based epigenetic markers are already under clinical evaluation for early detection of cancer (Xu et al., 2017; Oussalah et al., 2018). Additionally, the analysis of placenta-specific cf-DNA/RNA during early pregnancy could also be used for detection of adverse pregnancy outcomes prior to appearance of specific clinical features (Del Vecchio et al., 2020). Recent studies suggest that obesity may influence the changes in DNA methylation (Feinberg et al., 2010; Xu et al., 2013; Dick et al., 2014) which could possibly predict the future development of metabolic diseases. A genome-wide DNA methylation study in offsprings of women with high pre-pregnancy maternal BMI and gestational diabetes mellitus (GDM) identified 76 differentially methylated CpGs including several genes which are known to be associated with metabolic diseases. The study suggested that the methylation changes in the circulating blood cells could serve as a biomarker for prediction of metabolic diseases in offsprings of women with obesity and GDM (Hjort et al., 2018). A different study identified the differential methylation status of circulating cell-free CHTOP and INS1 DNA fragments as potential biomarkers for possible islet death in youths with obesity and diabetes (Syed et al., 2020). A study by Nishimoto et al. investigated the role of cfDNA in development adipose tissue inflammation. The study demonstrated that obesity induced cfDNA release from adipocytes promoted macrophage accumulation in the adipose tissue via TLR9 (Nishimoto et al., 2016). This novel mechanism for the development of adipose tissue inflammation may provide therapeutic target for obesity related metabolic disorders. Since the cell-free epigenetic markers are non-invasive, they may consequently be of greater clinical relevance for better prediction of metabolic disorders.

Histone acetylation and methylation are two of the most important epigenetic changes that regulate gene expression. Targeting these chromatin modifiers using small molecules and inhibitors has huge potential in treating obesity. HDAC inhibitors such as sodium butyrate and Trichostatin A, significantly decreased body weight in DIO mice (Gao et al., 2009). Other inhibitors targeting DNMTs, protein arginine methyltransferases, HDMs, and HATs are widely studied and have great potential in treating obesity, if used in systemic and strategic manner. However, the side effects and collateral damages caused by them due to their involvement in other cellular processes cannot be neglected thereby making their use challenging. Drug engineering for their controlled release and to enhance their specificity, could potentially reduce the side effects and toxicity. Some drugs and their modes of action in treating obesity are listed in Table 2.

**TABLE 2:** Drugs and their modes of action in treatment of obesity.

**Drug**	**Mode of Action**	**Status**	**Side effects**	**References**
**Metreleptin**	Activates OB receptor in peripheral tissues	Phase II	Headache, low blood sugar, abdominal pain, and dizziness	Heymsfield et al., 1999
**Orlistat (Xenical^®^)**	Pancreatic lipase inhibitor	EMA, FDA, ANVISA	Flatulence, oily stool, frequent bowel movement	Harp, 1999
**Naltrexone/bupropion (Contrave^®^)**	Opioid receptor antagonist/Noradrenaline and dopamine reuptake inhibitor	EMA, FDA	Nausea, constipation and headache	*NCT01601704
**Topiramate (Topamax^®^)**	Inhibits excitatory glutamate receptors and carbonic anhydrase	Phase II	Tiredness, drowsiness, coordination problems	*NCT01859013
**Phentermine (Adipex^®^)**	Noradrenergic sympathomimetic amine	EMA, FDA	Dizziness, dry mouth, insomnia, constipation, irritability and cardiovascular side effects	Hendricks et al., 2011
**Phentermine/topiramate (Qsymia^®^)**	Release of catecholamines and inhibits excitatory glutamate receptors and carbonic anhydrase	FDA	Paraesthesia, change in taste (dysgeusia) and metabolic acidosis	Allison et al., 2012
**Sibutramine (Biomag^®^, Sibus^®^, Saciette^®^)**	Inhibits 5-HT and norepinephrine reuptake	ANVISA	high blood pressure, shortness of breath	James et al., 2010
**Rimonabant (Acomplia^®^, Redufast^®^)**	Inverse agonist on the cannabinoid receptor CB1	Withdrawn after phase III	Nausea, diarrhea, and dizziness	*NCT00481975
**Lorcaserin (Belviq^®^)**	Serotonin receptor agonist	Withdrawn after phase III	Headache, dizziness, nausea, dry mouth, constipation, and increased risk of cancer	*NCT03353220
**Liraglutide (Victoza Saxenda^®^)**	GLP-1 receptor agonist	EMA, FDA, ANVISA	Nausea with vomiting are the principal adverse effects; acute pancreatitis	Gough, 2012
**Empagliflozin (Jardiance^®^)**	Sodium–glucose cotransporter 2 inhibitor	Phase I	Hypoglycemia, urinary problem	*NCT02798744
**Cetilistat (Cetislim^®^)**	Inhibits pancreatic lipase	Phase II	Loose stools, fecal incontinence and frequent bowel movements	Gras, 2013
**Beloranib**	Inhibitor of methionine aminopeptidase 2	Phase II and III	Diarrhea, abdominal pain	*NCT02324491

*Information retrieved from clinicaltrials.gov.

## Concluding Remarks

The past two decades of research in adipose biology made us acquainted with the fact that adipose tissue is not mere inert depot for fat storage, but is a highly complex and biologically active organ which plays vital roles in whole body energy metabolism and various physiological processes. The cooperative interplay between different transcription factors, specifically PPARγ and C/EBPα, is critical for understanding adipogenesis at molecular level. Any defects in the adipose function or adipogenesis process may result in severe metabolic abnormalities. Sometimes, genetic and acquired defects like familial lipodystrophy and diet induced obesity may also result in IR and diabetes. Therefore, understanding the heterogeneity and plasticity of adipose tissue is utmost important for targeting them to reap therapeutic benefits. Adipose tissue also serves as an endocrine organ and secretes many adipokines which associate them with different cancers. Apart from adipokines, they also secrete batokines which have been shown to improve insulin sensitivity and glucose tolerance. Thus, precise selection of batokines could serve the purpose of identifying candidates for drug development and ameliorating metabolic disorders (Villarroya et al., 2017). In recent years, there have been a number of clinical trials with anti-inflammatory agents in targeting obesity related metabolic diseases. However, none of them met the approval criteria due to small cohort size and shorter period of the trials (Mclaughlin et al., 2017). The ominous connection between epigenetic changes and environmental factors contributes largely to adult onset of obesity and metabolic disorders. Therefore, targeting epigenetic modulators using inhibitors and small molecules holds a great potential in treating obesity but their limited clinical efficacy and certain unavoidable side-effects make them difficult to use. Pharmacological therapy is used as an add-on anti-obesity therapy for the patients who fail to respond to lifestyle modifications. Some drugs, though successful, have variable response rates attributing to the individual variations. Therefore, future pharmacotherapy may include the use of personalized drugs to target obesity at individual level.

## Author Contributions

SC, RP, and AA contributed to conception of idea. RP, PF, VS, and AA contributed to manuscript writing. All authors contributed to manuscript editing.

## Conflict of Interest

The authors declare that the research was conducted in the absence of any commercial or financial relationships that could be construed as a potential conflict of interest.

## Footnotes

1 https://www.cancer.gov/about-cancer/causes-prevention/risk/obesity/obesity-fact-sheet

2 https://www.who.int/news-room/fact-sheets/detail/obesity-and-overweight

3 https://clinicaltrials.gov/
